# Using online databases to produce comprehensive accounts of the vascular plants from the Brazilian protected areas: The Parque Nacional do Itatiaia as a case study

**DOI:** 10.3897/BDJ.8.e50837

**Published:** 2020-05-19

**Authors:** Marina M Moreira, Tatiana T Carrijo, Anderson Alves-Araújo, André M A Amorim, Alessandro Rapini, Andrews V S da Silva, Braz A P Cosenza, Claudia R Lopes, Camila N Delgado, Cíntia Kameyama, Dayvid R Couto, Daniel E F Barbosa, Daniele Monteiro, Diego R Gonzaga, Eduardo C Dalcin, Elsie F Guimarães, Elton John de Lírio, Fernando B Matos, Fátima R G Salimena, Felipe A Oliveira, Gustavo Heiden, João M Lanna, José Fernando Baumgratz, José F B Pastore, Juliana R P M Oliveira, Laísa B Barcelos, Lana S Sylvestre, Leandro Freitas, Leandro L Giacomin, Leandro Pederneiras, Leonardo D Meireles, Lúcia G Lohmann, Luciana C Pereira, Luis Alexandre E Silva, Luiz M Neto, Marcelo C Souza, Marcelo Trovó, Marcos E G Sobral, Mário Luís Garbin, Mario Gomes, Marli P Morim, Michelle Christine A Mota, Paulo H Labiak, Pedro L Viana, Pedro Luís R de Moraes, Renato Goldenberg, Rubens Luiz G Coelho, Samyra G Furtado, Sebastião José da Silva-Neto, Thiago B Flores, Valquíria F Dutra, Vinícius R Bueno, Rafaela C Forzza

**Affiliations:** 1 Universidade Federal do Espírito Santo, Vitória, Brazil Universidade Federal do Espírito Santo Vitória Brazil; 2 Jardim Botânico do Rio de Janeiro, Rio de Janeiro, Brazil Jardim Botânico do Rio de Janeiro Rio de Janeiro Brazil; 3 Universidade Estadual de Santa Cruz, Ilhéus, Brazil Universidade Estadual de Santa Cruz Ilhéus Brazil; 4 Universidade Estadual de Feira de Santana, Feira de Santana, Brazil Universidade Estadual de Feira de Santana Feira de Santana Brazil; 5 Universidade Federal do Rio de Janeiro, Rio de Janeiro, Brazil Universidade Federal do Rio de Janeiro Rio de Janeiro Brazil; 6 Universidade do Estado de Minas Gerais, Belo Horizonte, Brazil Universidade do Estado de Minas Gerais Belo Horizonte Brazil; 7 Universidade Federal de Juiz de Fora, Juiz de Fora, Brazil Universidade Federal de Juiz de Fora Juiz de Fora Brazil; 8 Universidade Federal de Alfenas, Alfenas, Brazil Universidade Federal de Alfenas Alfenas Brazil; 9 Instituto de Botânica, São Paulo, Brazil Instituto de Botânica São Paulo Brazil; 10 Universidade Estadual do Norte Fluminense, Campos dos Goytacazes, Brazil Universidade Estadual do Norte Fluminense Campos dos Goytacazes Brazil; 11 Universidade de São Paulo, São Paulo, Brazil Universidade de São Paulo São Paulo Brazil; 12 Universidade Federal do Paraná, Curitiba, Brazil Universidade Federal do Paraná Curitiba Brazil; 13 Embrapa Clima Temperado, Pelotas, Brazil Embrapa Clima Temperado Pelotas Brazil; 14 Universidade Federal de Santa Catarina, Florianópolis, Brazil Universidade Federal de Santa Catarina Florianópolis Brazil; 15 Universidade Federal de Pelotas, Pelotas, Brazil Universidade Federal de Pelotas Pelotas Brazil; 16 Universidade Federal do Oeste do Pará, Santarém, Brazil Universidade Federal do Oeste do Pará Santarém Brazil; 17 Universidade Federal Rural do Rio de Janeiro, Seropédica, Brazil Universidade Federal Rural do Rio de Janeiro Seropédica Brazil; 18 Universidade Federal de São João Del-Rei, São João Del-Rei, Brazil Universidade Federal de São João Del-Rei São João Del-Rei Brazil; 19 Museu Paraense Emílio Goeldi, Belém, Brazil Museu Paraense Emílio Goeldi Belém Brazil; 20 Universidade Estadual Paulista Júlio de Mesquita Filho, São Paulo, Brazil Universidade Estadual Paulista Júlio de Mesquita Filho São Paulo Brazil; 21 The Flowr Corporation, Markham, Canada The Flowr Corporation Markham Canada; 22 Universidade do Estado do Rio de Janeiro, Rio de Janeiro, Brazil Universidade do Estado do Rio de Janeiro Rio de Janeiro Brazil; 23 Universidade Estadual de Campinas, Campinas, Brazil Universidade Estadual de Campinas Campinas Brazil; 24 Universidade Federal do Rio Grande do Sul, Porto Alegre, Brazil Universidade Federal do Rio Grande do Sul Porto Alegre Brazil

**Keywords:** Atlantic Forest, angiosperms, gymnosperms, lycophytes, ferns, plant collection.

## Abstract

**Background:**

Brazil is one of the most biodiverse countries in the world, with about 37,000 species of land plants. Part of this biodiversity is within protected areas. The development of online databases in the last years greatly improved the available biodiversity data. However, the existing databases do not provide information about the protected areas in which individual plant species occur. The lack of such information is a crucial gap for conservation actions. This study aimed to show how the information captured from online databases, cleaned by a protocol and verified by taxonomists allowed us to obtain a comprehensive list of the vascular plant species from the "Parque Nacional do Itatiaia", the first national park founded in Brazil. All existing records in the online database JABOT (15,100 vouchers) were downloaded, resulting in 11,783 vouchers identified at the species level. Overall, we documented 2,316 species belonging to 176 families and 837 genera of vascular plants in the "Parque Nacional do Itatiaia". Considering the whole vascular flora, 2,238 species are native and 78 are non-native.

**New information:**

The "Parque Nacional do Itatiaia" houses 13% of the angiosperm and 37% of the fern species known from the Brazilian Atlantic Forest. Amongst these species, 82 have been cited as threatened, following IUCN categories (CR, EN or VU), seven are data deficient (DD) and 15 have been classified as a conservation priority, because they are only known from a single specimen collected before 1969.

## Introduction

Brazil is one of the countries with the highest number of plant species in the world (Forzza et al. 2012). Overall, Brazil hosts about 37,000 species of land plants distributed through six phytogeographical domains (Flora do Brasil 2020 under construction 2019): Atlantic Forest, Amazon, Cerrado, Caatinga, Pampa and Pantanal. Amongst these domains, the Brazilian Atlantic Forest has the greatest species richness (BFG 2018), also representing a biodiversity hotspot (Myers et al. 2000). Over the last 80 years, 2,446 protected areas (Full protection: 777; Sustainable use: 1,669) were created in Brazil for biodiversity conservation (MMA 2020). Nowadays, there are 488 federal protected areas within the Atlantic Forest domain, corresponding to 20% of the existing conservation units in Brazil (MMA 2020). These protected areas are responsible for the defence and management of large reservoirs of biodiversity, carbon stocks and water, with substantial social and economic impact for the country (Hassler 2005, Medeiros et al. 2011). However, the knowledge about the flora within such protected areas is still dispersed in literature. Data access is thus very difficult for professionals who study the biodiversity within these areas, as well as those in charge of protecting and preserving this important biodiversity.

The list of species of the Brazilian Flora and the “Flora do Brasil 2020” (Forzza et al. 2010, BFG 2015, BFG 2018), the Red Book of the Brazilian Flora (Martinelli and Moraes 2013) and the Reflora Virtual Herbarium (Canteiro et al. 2019) considerably improved our knowledge about plant species richness and conservation in Brazil. Moreover, there has been a comprehensive advance in the online publication of biodiversity data in the last decades, promoted by the development of biodiversity information systems (e.g. PortalBio (2020), SiBBr (2020), Reflora (2020), INCT Herbário Virtual da Flora e dos Fungos (2020), GBIF (2020), CNCFlora (2020)). However, each one of these systems has been designed for specific purposes. For example, the “Centro Nacional para Conservação da Flora” (CNCFlora, National Center for Conservation of the Flora) provides a continuously updated list of threatened plants in the country (MMA 2014). None of these systems provides specialised functions or filters that allow us to retrieve information about plant species occurring within a given protected area. As a result, it is impossible to know how many and which plant species occur within each Brazilian protected area.

The “Parque Nacional do Itatiaia” (PNI, Itatiaia National Park) was founded in 1937, representing the first national park of Brazil (ICMBio 2013a). This federally-protected area covers about 30,000 ha between the states of Rio de Janeiro and Minas Gerais. The region where the PNI is located has been the focus of several scientific expeditions since the 19th century (see Morim 2006). Throughout that century, more than 50 Brazilian and European scientists collected plants and enriched herbarium collections with specimens from different locations in this important mountain complex (Urban 1906, Brade 1956, Mendes-Júnior et al. 1991).

Auguste François Marie Glaziou was the first botanist to visit the highest portions of Itatiaia (Brade 1956). However, the most significant contribution on Itatiaia’s flora was provided by Ernst Ule, who described the altitudinal zonation of the Park's vegetation back in the 19th century (Ule 1896). In the early 20th century, Per Karl Hjalmar Dusén provided descriptions and illustrations of plant species and considerations about their distribution in Itatiaia (Dusén 1903, Dusén 1955). Another important study conducted in the first half of the 20th century was a botanical survey conducted by Alexander Curt Brade, who published taxonomic treatments for several plant families in the region, as well as lists of species and endemism records, descriptions of phytophysiognomies and interpretations about the origin of the local flora (Brade 1956).

The flora of Itatiaia, published by Barroso et al. (1957), included taxonomic treatments for 20 plant families. Several taxonomic accounts of selected plant genera or families from Itatiaia were also published during the last 15 years (e.g. Lima and Guedes-Bruni 2004, Morim 2006, Morim and Barroso 2007, Monteiro and Guimarães 2008, Monteiro and Guimarães 2009, Ramos and Sylvestre 2010, Mezabarba et al. 2013, Giannerini et al. 2015, Rollim and Trovó 2016, Freitas et al. 2017, Gonzaga et al. 2017). Other studies focused on associations between climate and elevation (e.g. Segadas-Vianna 1965, Segadas-Vianna and Dau 1965), diversity and conservation of various plant groups (e.g. Condack and Sylvestre 2009, Costa et al. 2017, da Costa et al. 2015) and biogeography (e.g. Safford 1999a, Safford 1999b, Ribeiro and Medina 2002). All these studies helped to increase the number of plant specimens collected in the PNI and housed at various Brazilian herbaria. Most of these collections are deposited at the herbarium of the “Jardim Botânico do Rio de Janeiro” (RB, an acronym from Thiers, continuously updated) (Lanna et al. 2018). The digitisation of Brazilian herbaria allowed several databases to be published online in recent years. Despite the great efforts to make biodiversity information freely available, the data currently available is still not sufficient to allow the preparation of species lists for protected areas or lists of threatened taxa.

The databases, built over the course of those studies, allow anyone to easily access information on Brazilian plant species collections. This study aims to show how we can build a list of species from an important conservation unit from data recovered from online databases. We further illustrate the additional types of information that can be recovered from such datasets, including information on vegetation types, life forms, endemism, conservation status and number of herbarium records (Forzza and Lanna 2019). We further summarise information on threatened species status (CR, EN and VU categories) available from other resources. We also list the species that should be considered a priority for conservation policy, i.e. those collected more than 50 years ago and known from a single record in our database.

## Sampling methods

### Study extent

A list of all vascular plant specimens from PNI was downloaded from the database JABOT (“Jardim Botânico do Rio de Janeiro”, JBRJ, Botanical Garden of Rio de Janeiro, www.jbrj.gov.br/jabot; Silva et al. 2017). We performed three searches on 21 March 2018, using the following filters: (1) group = angiosperms, locality = Itatiaia; (2) group = gymnosperms, locality = Itatiaia; and (3) group = lycophytes and ferns, locality = Itatiaia. These searches led to a total of 15,100 records (12,786 angiosperms, 10 gymnosperms and 2,304 lycophytes and ferns).

**Figure 1.**
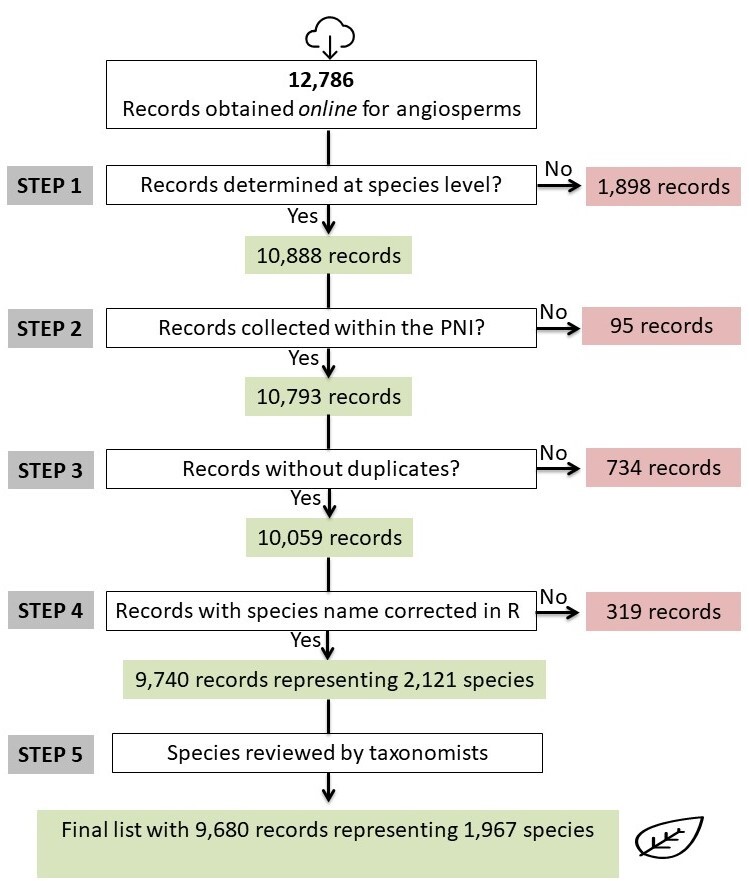
Stages of data cleaning performed in R to obtain a list of angiosperm species from the “Parque Nacional do Itatiaia,” Brazil, from the RB herbarium database. The specimens kept on the list are shown in green, while the specimens removed are shown in red.

**Figure 2.**
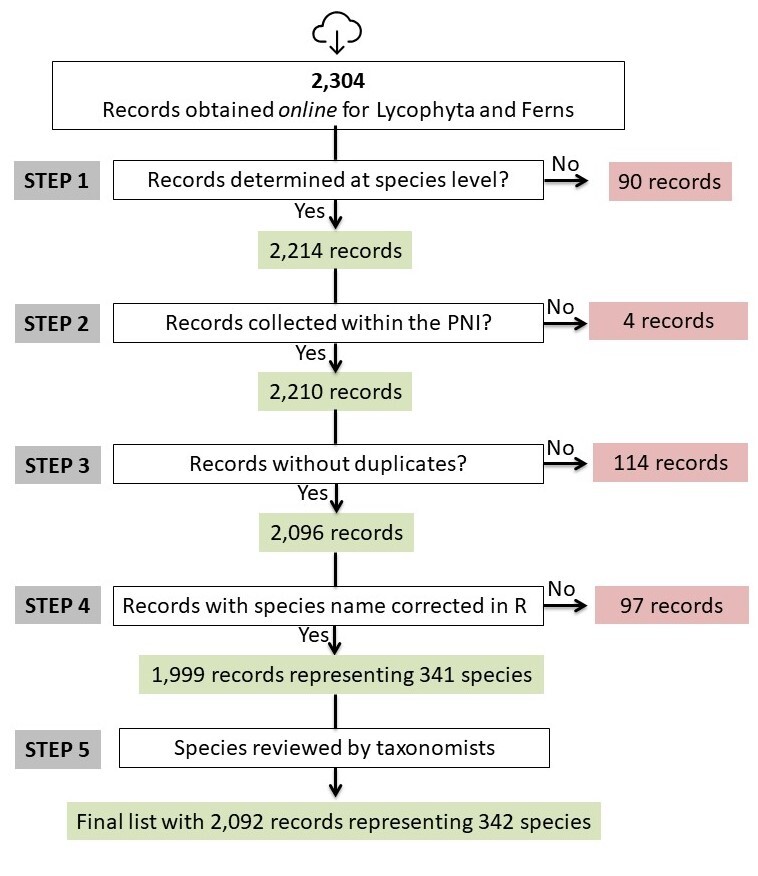
Stages of data cleaning performed in R to obtain a list of lycophyte and fern species from the “Parque Nacional do Itatiaia,” Brazil, from the RB herbarium database. The specimens kept on the list are shown in green, while the specimens removed are shown in red.

Based on these online records, we created a protocol (Figs 1, 2) to clean the database, in order to obtain a list of species with currently accepted nomenclature. The **first step** of this protocol was to manually select all records determined at the species level, which led to the following results: angiosperms (determined = 10,888; undetermined = 1,898), gymnosperms (determined = 6; undetermined = 4) and lycophytes and ferns (determined = 2,214; undetermined = 90; Figs 1, 2). Since only 10 records were recovered for gymnosperms, these records were analysed manually. We proceeded to the following steps using the software R, v. 3.4.4 (R Development Core Team 2018).

The subsequent steps performed in R were as follows: **(step 2)** removal of records in which the locality did not belong to the area covered by the PNI (angiosperms = 95 records removed; gymnosperms = 1; lycophytes and ferns = 4); **(step 3)** removal of duplicates (angiosperms = 734 records; gymnosperms = 0; lycophytes and ferns = 114). Duplicates were removed from the list based on collector name, collector number and the year in which the sample was collected. After removing all duplicates, 10,059 records for angiosperms, nine for gymnosperms and 2,096 for lycophytes and ferns remained in the list (Figs 1, 2). We then updated and corrected the species names and defined the threat categories (**step 4**; Figs 1, 2). We used the R function *get.taxa* from the flora package to correct and update species names (Carvalho 2017). This function compares the names in our list with those in the Brazilian Flora online list (Flora do Brasil 2020). Introduced species included in the Brazilian Flora were not recovered by the function *get.taxa* (angiosperms = 319 records; lycophytes and ferns = 97); those records were reviewed manually (Figs 1, 2). After these corrections, a preliminary list with 2,121 species of angiosperms and 341 species of lycophytes and ferns was generated and the names and identifications were subsequently checked by taxonomists (**step 5**; Figs 1, 2). When plant species names were modified by a taxonomist, at least one specimen of that particular species was updated in the RB herbarium database. After reviewing all names, taxonomists included additional records of species known to occur in the PNI, but only documented in other databases (e.g. INCT Herbário Virtual da Flora e dos Fungos, Herbário Virtual Reflora). At this stage, specimens collected outside PNI were removed; because the R protocol failed to remove several records due to digitisation errors, this process had to be conducted manually. After all these steps, our final list included 11,783 records from the PNI (angiosperms = 9,680 records; gymnosperms = 11; lycophyte and ferns = 2,092; Figs 1, 2; Suppl. materials 1, 2).

**Vegetation types and life forms**: We obtained information on vegetation type and life form from the Brazilian Flora (http://floradobrasil.jbrj.gov.br) for every species included in the clean list. Although the PNI encompasses several different vegetation types, we classified all species as either occurring in forests (all forest types) or grasslands (all types of grasslands and inselbergs) or both. We chose this broader classification system because many records did not have detailed information about vegetation type to allow for finer scale classifications. Life forms were classified into five categories: trees, shrubs, sub-shrubs, lianas and herbs, based on information from the Brazilian Flora website (http://floradobrasil.jbrj.gov.br). When the Brazilian Flora provided more than one life form type for a given species, we chose the most frequent life form observed in the herbarium records for PNI (http://floradobrasil.jbrj.gov.br).

**Endemism and threatened species**: To evaluate whether species were native or non-native from Brazil, endemic or non-endemic to Brazil and to assign a threat category, we used information from the Brazilian Flora (http://floradobrasil.jbrj.gov.br) and CNCFlora (http://www.cncflora.jbrj.gov.br/portal), the Red List Authority for plants in Brazil. It should be noted that the vascular species list for PNI provided here does not include infraspecific taxa. In the case of species not included in the Brazilian Flora database, threat category and origin (native/non-native) were obtained from taxonomists. We considered as non-native, all species indicated as not occurring in Brazil or being cultivated or naturalised within the Brazilian Flora database.

**Priority species for conservation**: We classified a species as a priority for conservation, whenever it presented a single record collected before 1969 (Briggs and Leigh 1988) and was simultaneously categorised as critically endangered (CR), vulnerable (VU), endangered (EN) or data deficient (DD), according to CNC Flora (http://www.cncflora.jbrj.gov.br/portal).

## Geographic coverage

### Description

The "Parque Nacional do Itatiaia" (PNI) comprises the municipalities of Bocaina de Minas and Itamonte in the state of Minas Gerais and Itatiaia and Resende in the state of Rio de Janeiro, all within the Serra da Mantiqueira mountain range. The altitudinal range varies from 700 and 2,787 m, the latter at the summit of the “Pico das Agulhas Negras” (Fig. 3c). The climate is mesothermic, with an annual average temperature between 15º and 21ºC, depending on the elevation (ICMBio 2013b). The park can be accessed by two entrances: The Lower Portion, characterised by large waterfalls such as "Cachoeira Véu da Noiva" (Fig. 3a), "Maromba" and "Itaporani" (Fig. 3b) and the Upper Portion, which includes the formations of the "Agulhas Negras" (Fig. 3c), "Prateleiras" (Fig. 3d) and "Morro do Couto".

### Coordinates

-22º45' and -22º19' Latitude; -44°45' and -44°50' Longitude.

## Taxonomic coverage

### Description

We recorded 2,316 species of vascular plants for PNI, including native (2,238 species) and non-native (78) (Suppl. material 3). For angiosperms, we recorded 1,967 species (native = 1,899; non-native = 68) belonging to 143 families and 722 genera. The native species recorded here represent 6% and 13% of the angiosperms recognised for Brazil and the Atlantic Forest, respectively (Flora do Brasil 2020 under construction 2019). The ten richest families in the PNI (Fig. 4a) accounted for 54% (i.e. 1,059 species) of the total species. Seven of these families have also been reported as the ten richest angiosperm families for the Atlantic Forest (BFG 2015). Different from BFG (2015), Bromeliaceae, Apocynaceae and Euphorbiaceae are not amongst the ten richest families in the PNI. This is probably due to the great topographic heterogeneity of the Atlantic Forest (Nettesheim et al. 2018), which may not be represented in a single conservation unity. Thirty families are represented by a single species in the PNI. The ten richest genera accounted for 15% (i.e. 294 species) of the total species found in the PNI (Fig. 4b). In total, 391 genera are represented by a single species.

The seven species of gymnosperms documented in the PNI belong to three families, Araucariaceae (1 species), Cupressaceae (4) and Podocarpaceae (2) and six genera: *Araucaria*, *Cryptomeria*, *Cunninghamia*, *Cupressus*, *Thuja* (represented by one species each) and *Podocarpus* (represented by two species). All these species of gymnosperms are non-native, except for *Araucaria angustifolia* (Bertol.) Kuntze and *Podocarpus lambertii* Klotzsch ex Endl.

For lycophytes and ferns, we recorded 342 species (native = 337; non-native = 5) belonging to 30 families and 109 genera. The ten richest families in the PNI (Fig. 5a) accounted for 84% (286 species) of the total species. Ten families are represented by a single species. The ten richest genera in the PNI accounted for 41% (i.e. 139 species) of the total species (Fig. 5b). Amongst the genera, 57 presented a single species. The native species found in the PNI represent 24% and 37% of the lycophytes and ferns documented for Brazil and the Atlantic Forest, respectively (Flora do Brasil 2020 under construction 2019).

## Traits coverage

The PNI presents diverse environmental conditions (i.e. climatic, altitudinal and edaphic) and includes different vegetation types, such as high montane forests, seasonal rainforests ("florestas altomontanas estacionais semideciduais e ombrófilas densas”; see (Veloso et al. 1991), Araucaria forests (“florestas com araucária” or “floresta ombrófila mista”), high altitude grasslands (“campos de altitude” and “campos rupestres”) and inselbergs (Mello and Mello 1909, Várzea 1942, Machado-Filho et al. 1983, Meireles et al. 2014).

### Vegetation types and life forms

Amongst angiosperms, 73% (1,437) of the species are restricted to forests, 16% (320) are restricted to grasslands and 7% (136) occur in both forests and grasslands. We did not have information on vegetation type for 4% (74) of the angiosperm species recorded (Suppl. material 3). For gymnosperms, 57% (4) of the species are restricted to forests and we did not have information on vegetation type for 43% (3) of the species recorded. For lycophytes and ferns, 73% (249) of the species are restricted to forests, 16% (55) occur in forests and grasslands and 9% (32) are restricted to grasslands. We did not have information on vegetation type for 2% (6) of the lycophyte and fern species (Suppl. material 3).

**Figure 6.**
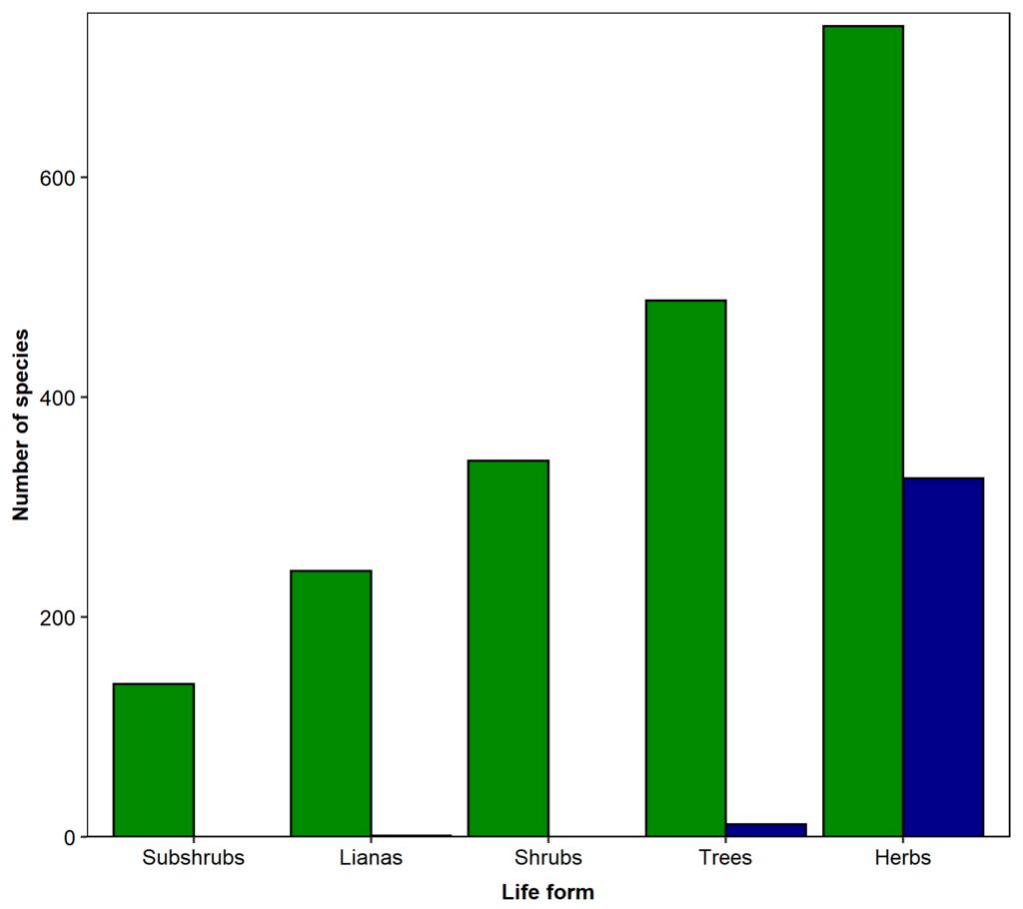
Life forms of the vascular plant species occurring in the “Parque Nacional do Itatiaia,” Brazil. The values within bars represent the number of species in each category. Green bars represent species of angiospem and blue bars represent species of lycophytes and ferns.

Overall, 38% (738 species) of all angiosperms recorded are herbs, followed by trees (25%; 488), shrubs (17%; 341), lianas (12%; 242) and subshrubs (7%; 139; Fig. 6; Suppl. material 3). Life form information was not available for 1% (19 species) of all species recorded (Suppl. material 3). All gymnosperms recorded are trees (Suppl. material 3). For lycophytes and ferns, 95.3% (326 species) of the species recorded are herbs, 3.2% (11) are trees and 0.3% (1) are lianas. We did not have information about life form for 1.2% (four species) of all species recorded (Fig. 6; Suppl. material 3).

### Endemism, conservation status, unique and old records

As far as the endemic species are concerned, 58% (1,140) of the angiosperms and 41% (140) of the lycophytes and ferns are endemic to Brazil. For gymnosperms, a single species is endemic (Suppl. material 3).

Amongst angiosperms, 66.2% (1,303 species) were recorded recently (i.e. after 1969), while 33.1% (650) have only old records; we did not have information about the collection year for 0.7% (14; Suppl. material 3). For gymnosperms, 29% (2) of the species have recent records, while 71% (5) have only old records (Suppl. material 3). The proportion of species with recent records was higher in lycophytes and ferns than other groups, with 76.9% (263) of all species presenting recent records, 22.8% (78) presenting old records exclusively and 0.3% (1) not presenting information on the collection year (Suppl. material 3).

Considering the species with unique records, we observed that 30% (597 species) of the angiosperms have a single record, 16% (315) have two records and 11% (226) have three records (Suppl. material 3). For gymnosperms, 71% (five species) have a single record (Suppl. material 3). For lycophytes and ferns, this proportion was lower than in the other groups, with 21% of the species (73) showing unique records (Suppl. material 3).

We recorded 73 species of angiosperms that have already been cited as threatened (Fig. 7), either as critically endangered (three species), endangered (41) or vulnerable (29) and seven species with deficient data (DD; Suppl. material 4). These species belong to 39 families, with the Orchidaceae showing the highest number of threatened species (10 species) followed by Asteraceae, Bromeliaceae and Myrtaceae (six species each), Fabaceae (four species), Cactaceae, Lauraceae, Poaceae, Rubiaceae, Symplocaceae and Xyridaceae (three species each), Piperaceae and Smilacaceae (two species each) and 26 families with a single species in the list (Suppl. material 4). A single gymnosperm, *Araucaria angustifolia*, is threatened (EN) (Suppl. material 4). We recorded eight threatened species within lycophytes and ferns (EN = 6 species; VU = 2) belonging to five families (Suppl. material 4). The endangered species of PNI represent 5% of the endangered Atlantic Forest flora (species evaluated = 3,595; endangered species = 1,544) and 3% (265) of the species classified as data deficient in the Brazilian Flora’s Red Book (Martinelli and Moraes 2013).

### Priority species for conservation in the PNI

We selected 15 angiosperm species as a priority for conservation in the PNI (Table 1). Amongst gymnosperms, only *Araucaria angustifolia* is a priority for conservation. Although we found a single record of *A. angustifolia* collected before 1969 in the RB database, this species is not rare in the PNI, as observed during fieldwork. However, we decided to maintain this species as a priority for conservation. We did not find any threatened species of lycophytes and ferns that fit the criteria here adopted for conservation priority. Indeed, all threatened species in this group have more than one recent record deposited at RB (except *Grammitis fluminensis*, which has a single recent record at RB; Suppl. material 3).

## Temporal coverage

### Notes

Amongst the 11,783 records from the PNI, 11,737 (99.6%) are from RB (Suppl. material 1), while 46 (0.4%) are from other herbaria (Suppl. material 2). The oldest angiosperm record housed at RB was a sample collected by Auguste François Marie Glaziou in 1871 (RB01181837, RB00084452; Suppl. material 1). For gymnosperms, the oldest record deposited at RB was a sample without the named collector from 1932 (RBcarpo00776085, Suppl. material 1). For lycophytes and ferns, the oldest record was a sample collected by Glaziou in 1871 (RB00640274; Suppl. material 1).

**Figure 8.**
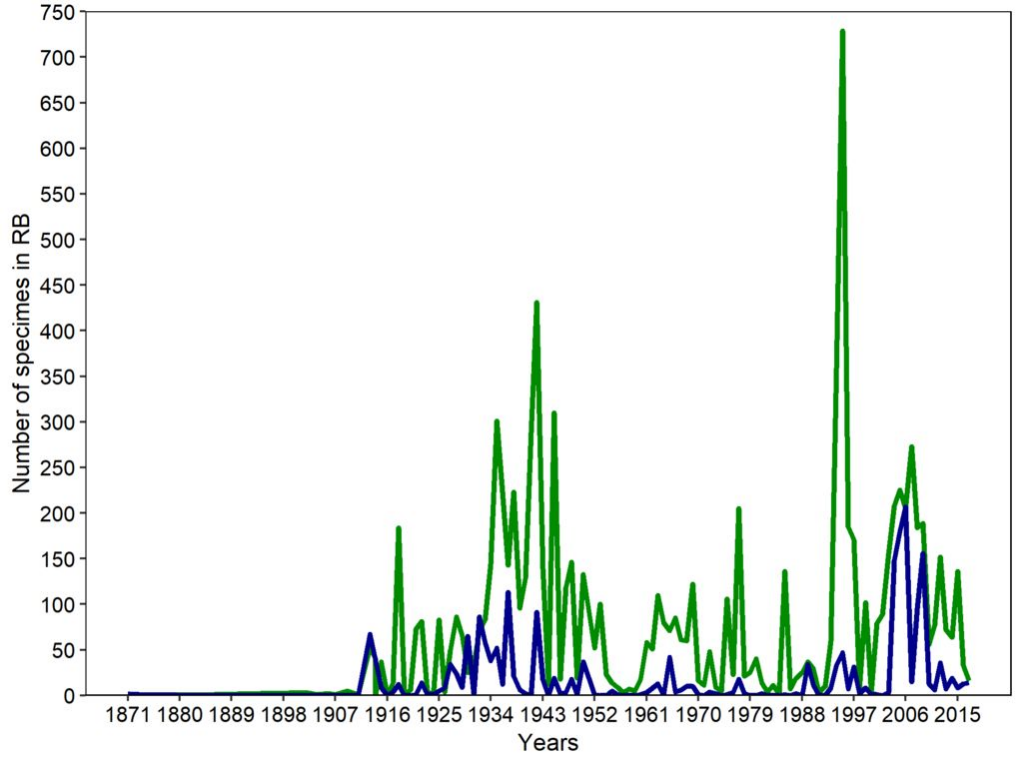
Number of specimens collected over time in the “Parque Nacional do Itatiaia” and housed in the RB herbarium. Green lines represent species of angiospem, while blue lines represent species of lycophytes and ferns.

The year with the highest number of collections of angiosperms deposited at RB was 1995 (729 records), followed by 1942 (431) and 1994 (359; Fig. 8). The large number of collections between 1994 and 1995 results from intense efforts conducted by the team of the "Programa Mata Atlântica" (PMA, Atlantic Forest Program). The PMA was coordinated by a group of researchers from JBRJ who surveyed several Atlantic Forest remnants in the state of Rio de Janeiro. The high number of records in 1942 was mostly due to collections by Alexander Curt Brade (Brade 1956). We did not obtain information on the collection year for 177 specimens (Suppl. material 1). For gymnosperms, we recorded a single sample for each year; one record did not include a collection date (Suppl. material 1). For lycophytes and ferns, the year with the highest number of records deposited at RB was 2006 (207 records), followed by 2005 (180), 2009 (156) and 2004 (148; Fig. 8). The high number of records between 2004 and 2009 was mostly due to collections by João Paulo Santos Condack (Condack 2006), Carla Gabriela Vargas Ramos and Lana da Silva Sylvestre (Ramos and Sylvestre 2010). The collection year was lacking for 18 records (Suppl. material 1).

The angiosperm records deposited at RB were collected by 294 collectors, while gymnosperm records were collected by eight collectors and lycophyte and fern records were collected by 85 collectors. For angiosperms, the collectors with more samples deposited at RB were Alexander Curt Brade (1,110 samples), João Marcelo Alvarenga Braga (953), Paulo de Campos Porto (849), Wanderbilt Duarte de Barros (698), Sócrates de Andrade (403), Sebastião da Silva Neto (348), Luiz Lanstyak (306), Edmundo Pereira (237), Felipe F. V. A. Barberena (189) and Gustavo Martinelli (186; Suppl. material 1). Collector name is missing in 132 records of angiosperms (Suppl. material 1). For gymnosperms, the main collectors were Sócrates de Andrade (2) and Wanderbilt Duarte de Barros (two samples); the other collectors collected a single sample (Suppl. material 1). One sample lacks the collector name (Suppl. material 1). For lycophytes and ferns, the main collectors in RB were Alexander Curt Brade (461 samples), Elaine Ribeiro Damasceno (240), João Paulo Santos Condack (231), Lana da Silva Sylvestre (226), Paulo de Campos Porto (194), João Marcelo Alvarenga Braga (78), Carla Gabriela Vargas Ramos (62), Roberto L. Cordeiro (59), Sócrates de Andrade (53) and Firmino Tamandaré de Toledo Júnior (53). Collector name is missing in 21 records (Suppl. material 1).

## Usage rights

### Use license

Open Data Commons Attribution License

## Data resources

### Data package title

Catálogo de Plantas das Unidades de Conservação do Brasil - Parque Nacional do Itatiaia (PNI)

### Resource link

http://ipt.jbrj.gov.br/jbrj/resource?r=catalogoucs

### Alternative identifiers

www.gbif.org/dataset/021cf0d3-aae6-417d-8682-ae535d17de89

### Number of data sets

1

### Data set 1.

#### Data set name

Catálogo de Plantas das Unidades de Conservação do Brasil - Parque Nacional do Itatiaia (PNI)

#### Data format

Darwin Core Archive

#### Number of columns

19

#### Download URL

http://ipt.jbrj.gov.br/jbrj/archive.do?r=catalogoucs&v=1.34

#### 

**Data set 1.** 

Column label	Column description
taxonID	The unique identifier for the Taxon.
scientificName	The full scientific name, including authorship.
kingdom	The full scientific name of the kingdom in which the taxon is classified.
family	The full scientific name of the family in which the taxon is classified.
genus	The full scientific name of the genus in which the taxon is classified.
specificEpithet	The name of the first or specific epithet of the scientificName.
taxonRank	The name of the lowest or terminal infraspecific epithet of the scientificName.
scientificNameAuthorship	The authorship information for the scientificName.
modified	The most recent date-time on which the resource was changed.
rightsHolder	A person or organisation owning or managing rights over the resource.
typeStatus	Status of the type. Controlled vocabulary of terms (holotype, lectotype, isotype, syntype, paratype, neotype, epitype, typus). The category "typus" is used for undefined type status.
taxonRank	The taxonomic rank of the detailed identification name in the scientificName.
collectionCode	The name, acronym, coden or initial identifying the collection or dataset from which the record was derived.
catalogNumber	Specimen barcode.
locality	Detailed description of the locality where a specimen was collected. Less specific geographic information can be provided in other geographic terms (higherGeography, continent, country, stateProvince, county, municipality, waterBody, island, islandGroup). This term may contain information modified from the original to correct perceived errors or to standardise the description.
recordedBy	A list (concatenated and separated) of names of people, groups or organisations responsible for recording the original occurrence.
EventDate	Date of collection.
verbatimLongitude	The geographic longitude (in decimal degrees, using the spatial reference system given in geodeticDatum) of the geographic centre of a locality. Positive values are east of the Greenwich Meridian, negative values are west of it. Legal values lie between -180 and 180, inclusive.
verbatimLatitude	The geographic latitude (in decimal degrees, using the spatial reference system given in geodeticDatum) of the geographic centre of a location. Positive values are north of the Equator, negative values are south of it. Legal values lie between -90 and 90, inclusive.

## Additional information

### Conclusions and prospects

The information captured from online databases, cleaned by a protocol and checked by taxonomists allowed us to build a comprehensive list of vascular plant species for the PNI that is available publicly through the site “Catálogo de Plantas das Unidades de Conservação do Brasil” (Catalogue of the Plants in Protected Areas of Brazil, http://ipt.jbrj.gov.br/jbrj/resource?r=catalogoucs). The catalogue provides prompt access to information on the PNI flora, indicating the importance of the park for the conservation of plant species from the Atlantic Forest of Brazil. The PNI list includes species that are scarcely represented in herbarium collections and species that are documented through a single herbarium specimen collected, as well as those that are endangered but occur in the PNI and species that are still poorly studied to ensure on-site conservation.

## Supplementary Material

2D267731-E12D-5EEB-9E92-C7FE3B7FA674 10.3897/BDJ.8.e50837.suppl1 Supplementary material 1 Records obtained from the online database, after the filtering protocol. Data type occurrences File: oo_367152.txt https://binary.pensoft.net/file/367152 Moreira et al.

EA68EE22-4BA8-5A7E-8CD9-F9BCF834070F 10.3897/BDJ.8.e50837.suppl2 Supplementary material 2 Records obtained from other online databases. Data type occurrences File: oo_367151.txt https://binary.pensoft.net/file/367151 Moreira et al.

E05C3B92-E46D-535E-B62A-4F8D7DC7542E 10.3897/BDJ.8.e50837.suppl3 Supplementary material 3 List of vascular plants occurring in the "Parque Nacional do Itatiaia" providing information on the number of specimens per species in the database, threat category, presence of old records (True = presence of only old records, False = presence of old and new records), life form, origin (native vs. non-native), endemism and vegetation type. No information = indicates that data is lacking for that species. Data type List of species File: oo_401424.txt https://binary.pensoft.net/file/401424 Moreira et al.

8F09A07A-C7FE-51CA-92AC-BA0EC44D0E3A 10.3897/BDJ.8.e50837.suppl4 Supplementary material 4 Threatened and data deficient species of vascular plants occurring in the “Parque Nacional do Itatiaia,” their respective groups, families and threat category (CR = critically endangered, VU = vulnerable, EN = endangered and DD = data deficient). Data type List of species File: oo_352210.txt https://binary.pensoft.net/file/352210 Moreira et al.

## Figures and Tables

**Figure 3a.**
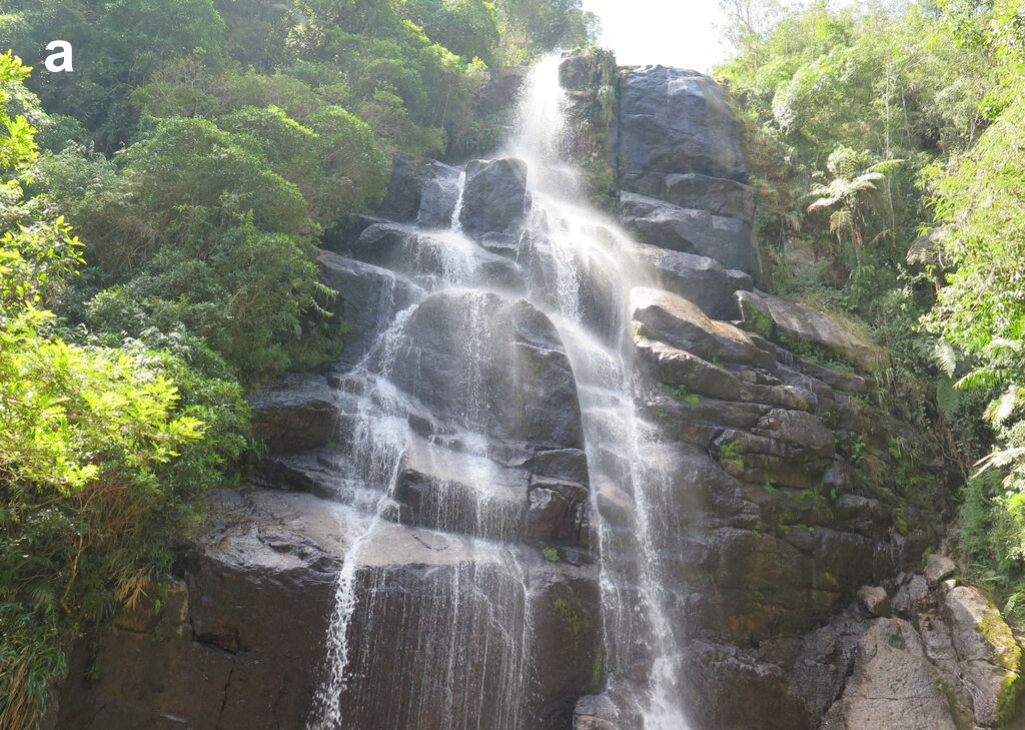
"Véu da Noiva" walterfalls

**Figure 3b.**
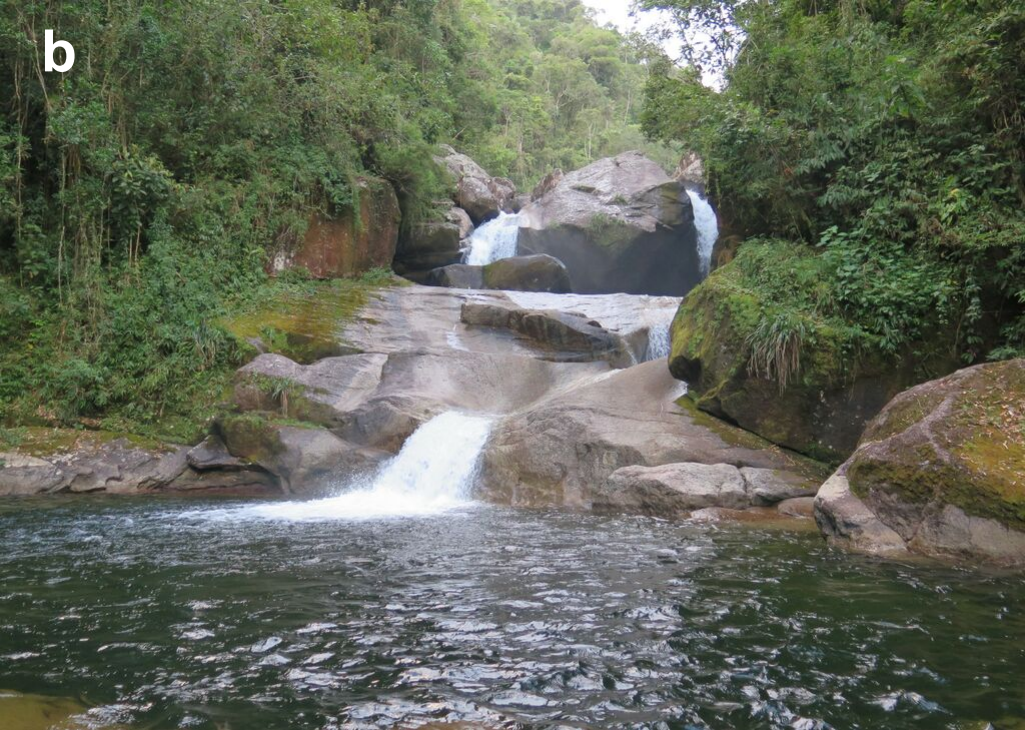
"Itaporani" walterfalls

**Figure 3c.**
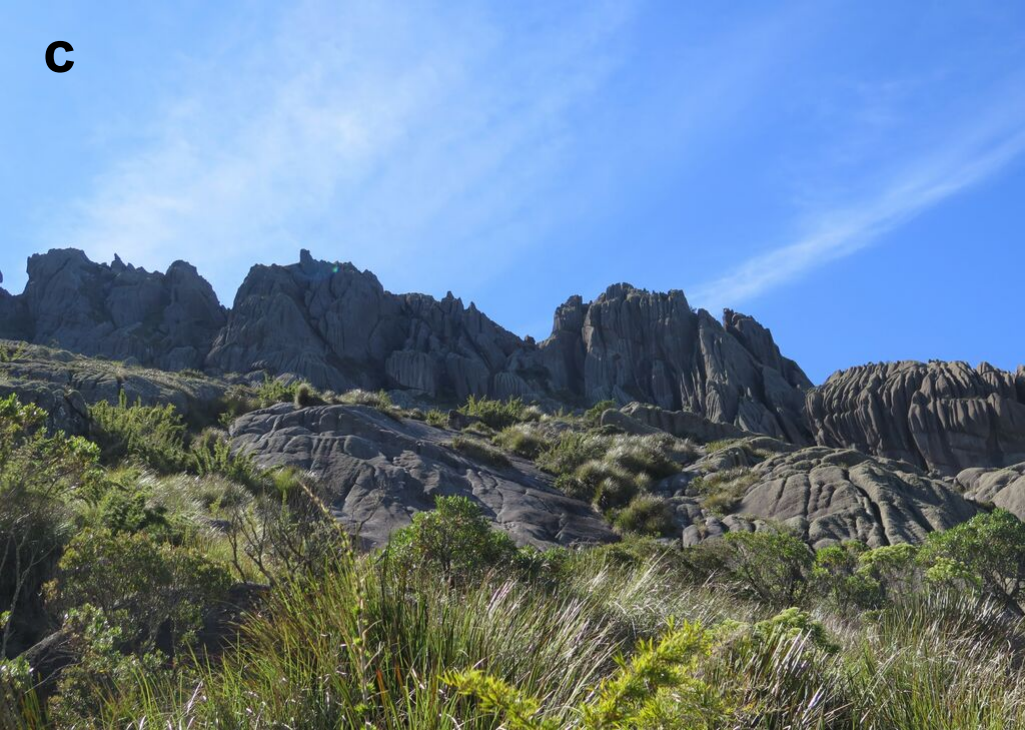
“Pico das Agulhas Negras”

**Figure 3d.**
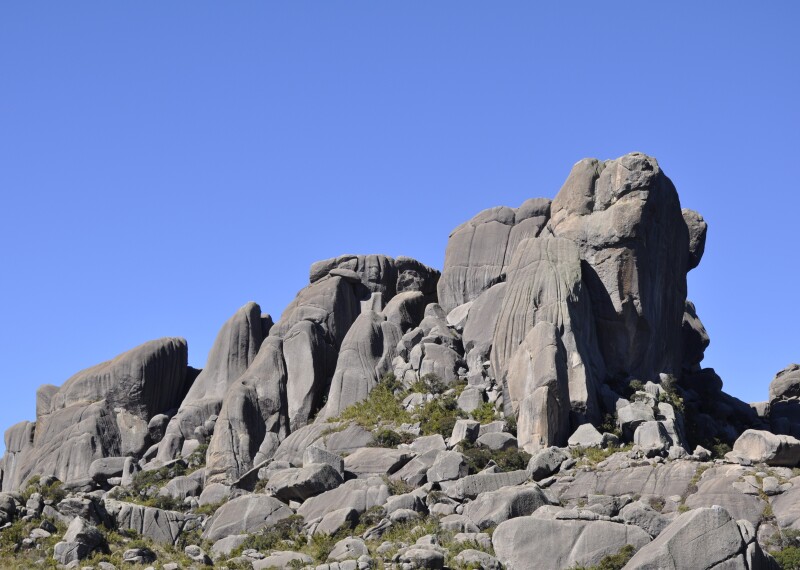
“Prateleiras”

**Figure 4a.**
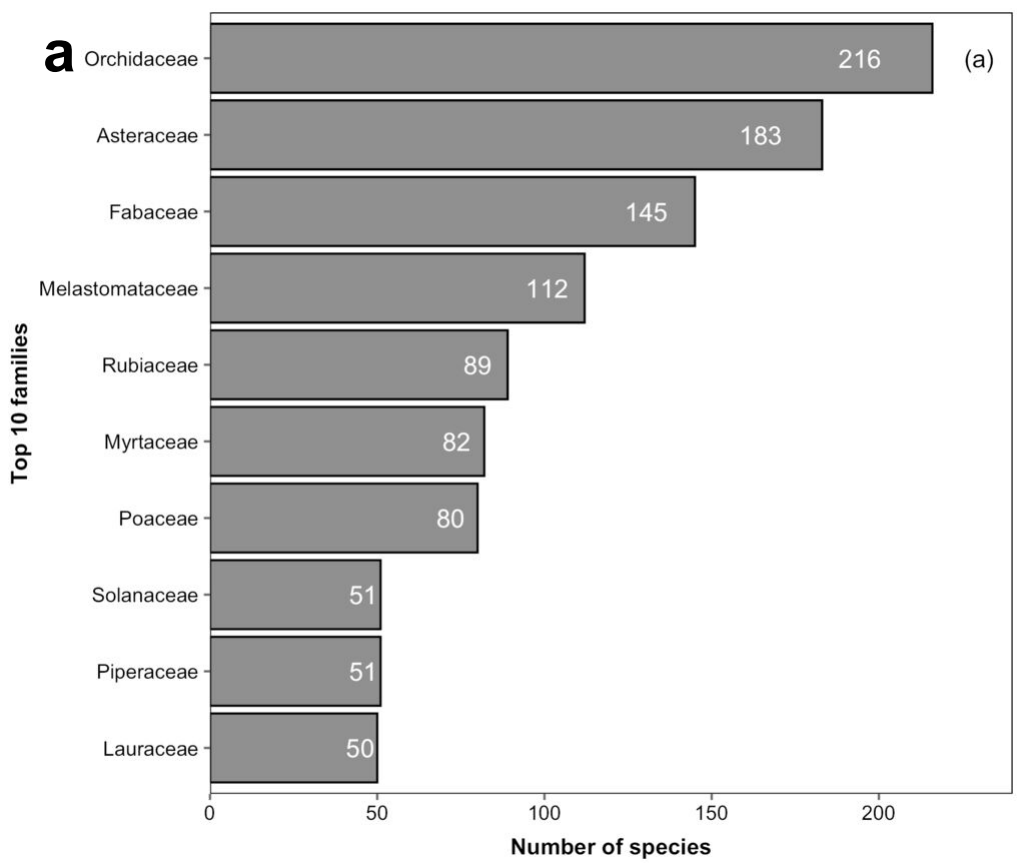


**Figure 4b.**
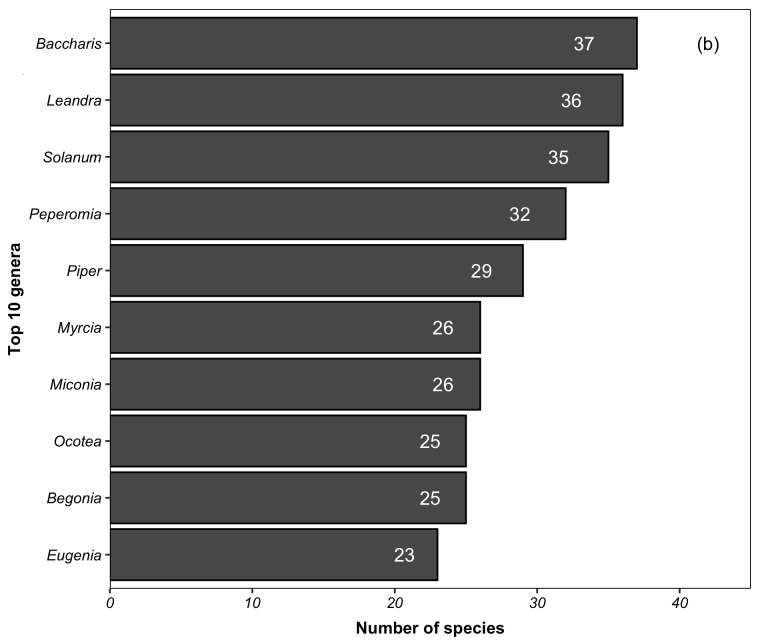


**Figure 5a.**
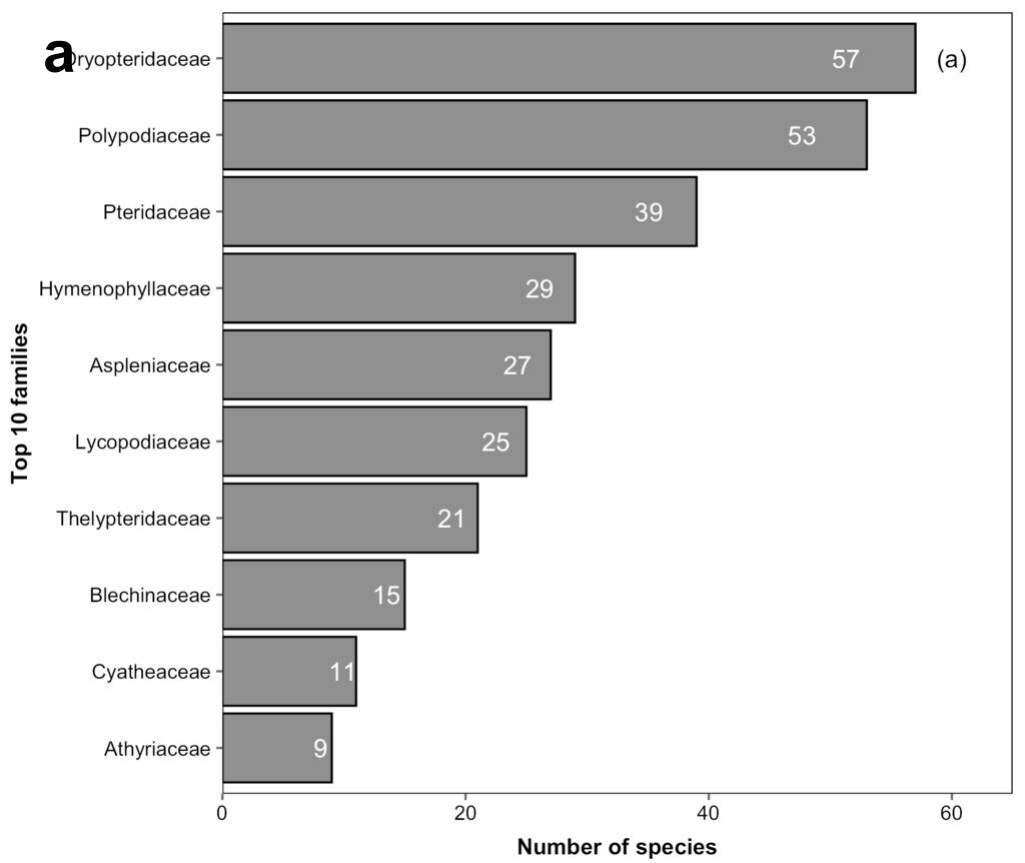


**Figure 5b.**
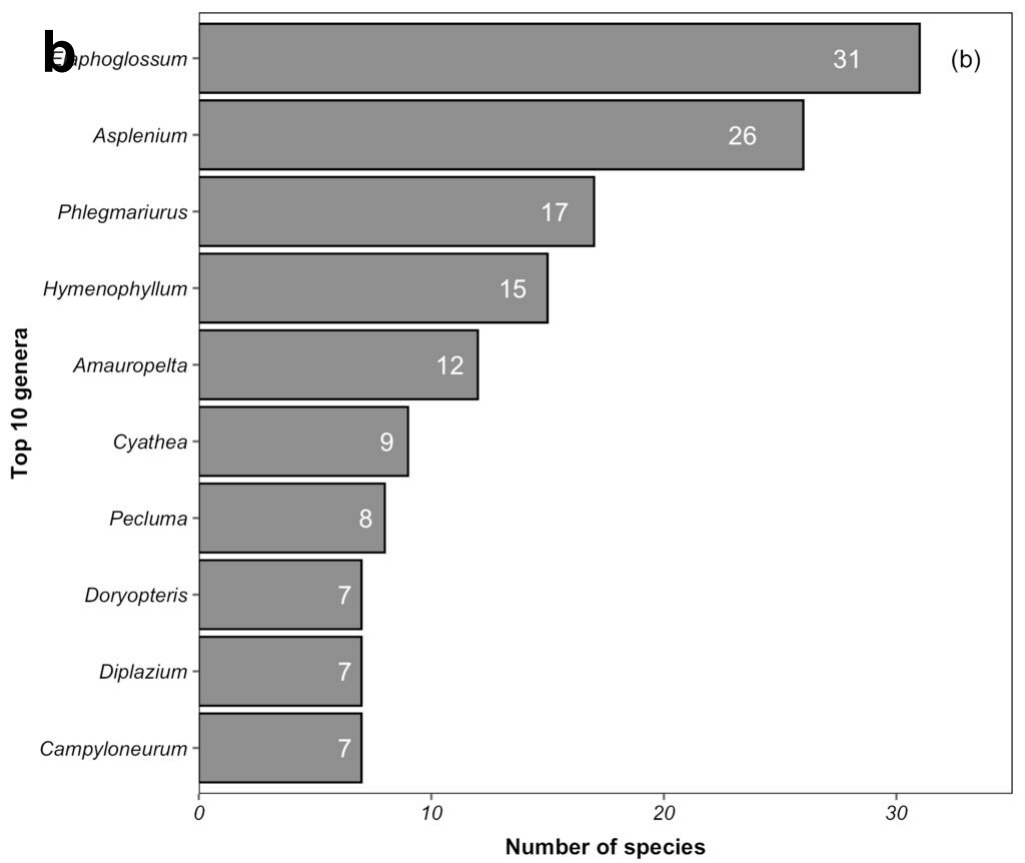


**Figure 7a.**
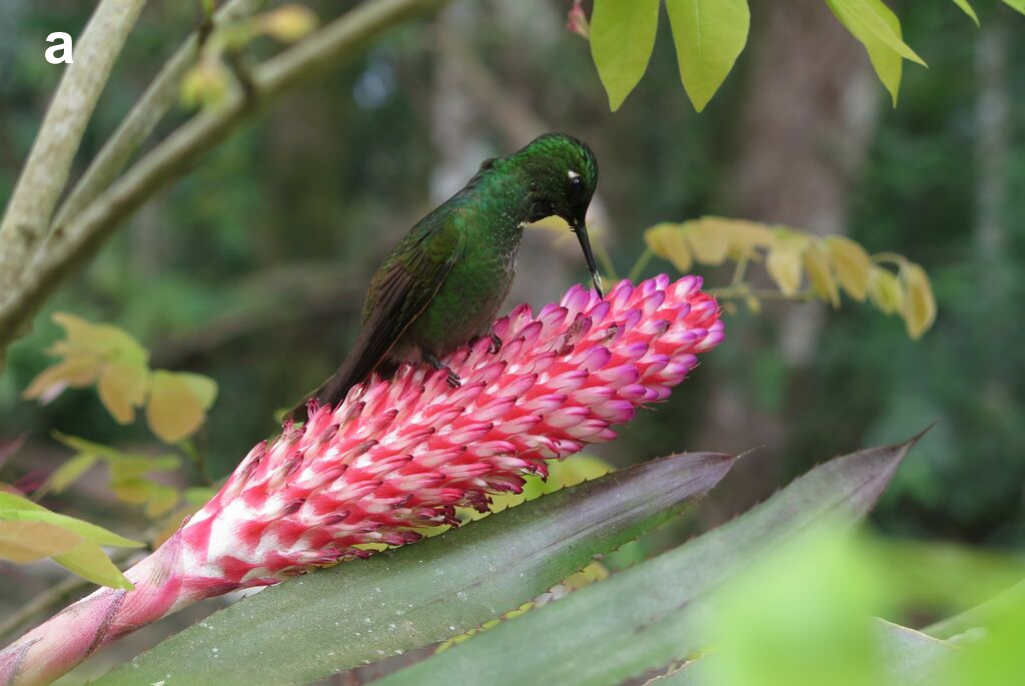
*Aechmea vanhoutteana* (Van Houtte) Mez - Vunerable (VU)

**Figure 7b.**
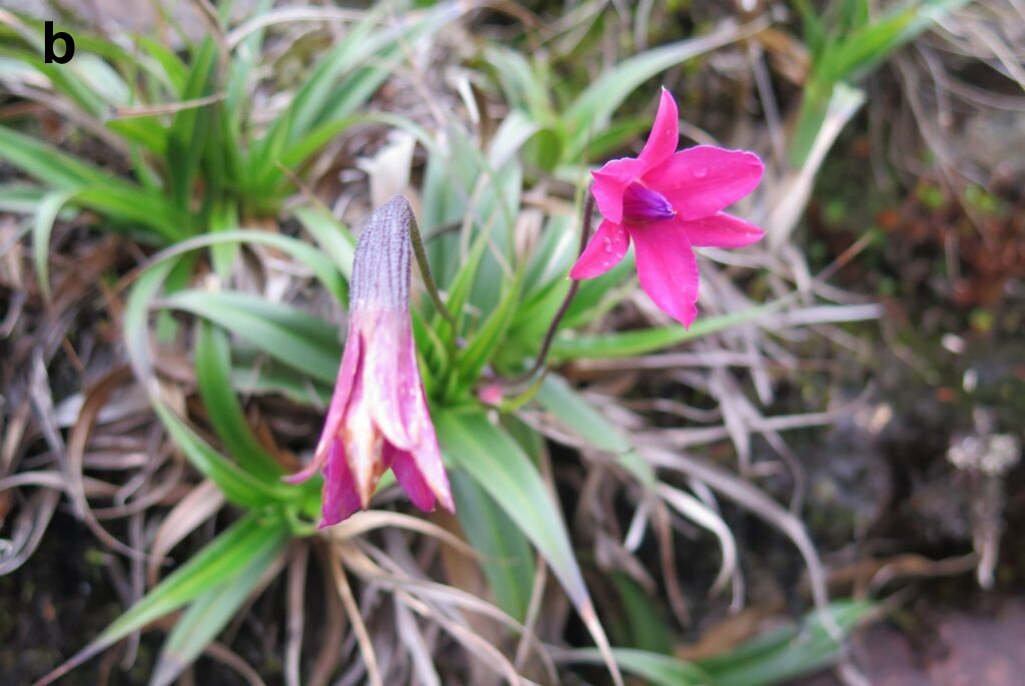
*Barbacenia gounelleana* Beauverd - Endangered (EN)

**Figure 7c.**
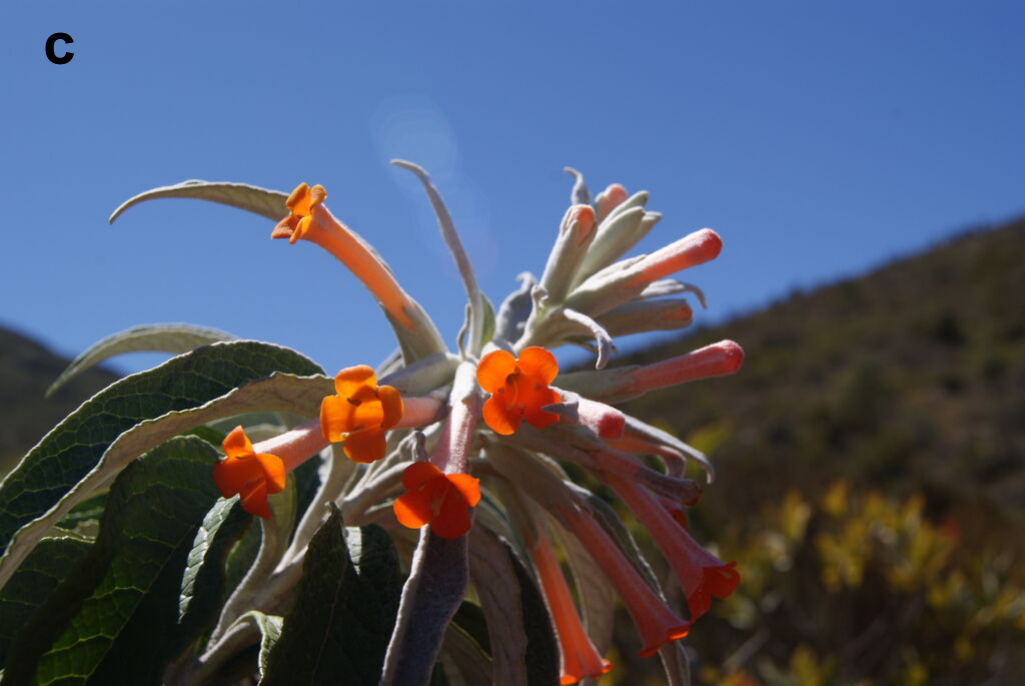
*Buddleja speciosissima* Taub. - Vunerable (VU)

**Figure 7d.**
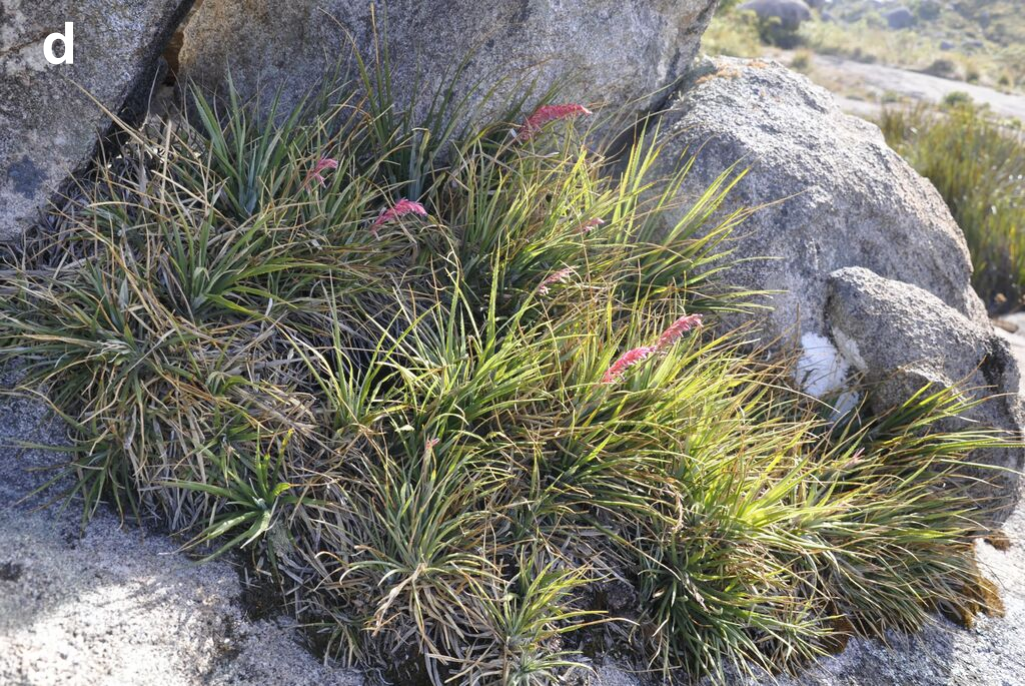
*Fernseea itatiaiae* (Wawra) Baker - Endangered (EN)

**Table 1.** List of species considered as a priority for conservation in the “Parque Nacional do Itatiaia”, Brazil. (CR = critically endangered, VU = vulnerable, EN = endangered and DD = data deficient), according to CNC Flora (http://www.cncflora.jbrj.gov.br/portal).

**Group**	**Family**	**Species**	**Category**
Angiosperms	Aquifoliaceae	*Ilex loranthoides* Mart. ex Reissek	VU
Angiosperms	Asteraceae	*Campuloclinium parvulum* (Glaz.) R.M.King & H.Rob.	VU
Angiosperms	Bromeliaceae	*Vriesea morrenii* Wawra	DD
Angiosperms	Bromeliaceae	*Vriesea sazimae* Leme	VU
Angiosperms	Chrysobalanaceae	*Licania indurata* Pilg.	EN
Angiosperms	Cyperaceae	*Cryptangium polyphyllum* (Nees) Boeckeler	EN
Angiosperms	Lauraceae	*Beilschmiedia rigida* (Mez) Kosterm.	EN
Angiosperms	Orchidaceae	*Anathallis tigridens* (Loefgr.) F.Barros & Barberena	VU
Angiosperms	Orchidaceae	*Grandiphyllum divaricatum* (Lindl.) Docha Neto	VU
Angiosperms	Orchidaceae	*Habenaria achalensis* Kraenzl.	VU
Angiosperms	Orchidaceae	*Isabelia virginalis* Barb.Rodr.	VU
Angiosperms	Orchidaceae	*Octomeria decumbens* Cogn.	DD
Angiosperms	Orchidaceae	*Pabstia jugosa* (Lindl.) Garay	EN
Angiosperms	Violaceae	*Viola gracillima* A.St.-Hil.	EN
Gymnosperms	Araucariaceae	*Araucaria angustifolia* (Bertol.) Kuntze	EN
